# Identification of Candidate Genes Controlling Soybean Cyst Nematode Resistance in “Handou 10” Based on Genome and Transcriptome Analyzes

**DOI:** 10.3389/fpls.2022.860034

**Published:** 2022-03-15

**Authors:** He Wei, Yun Lian, Jinying Li, Haichao Li, Qijian Song, Yongkang Wu, Chenfang Lei, Shiwei Wang, Hui Zhang, Jinshe Wang, Weiguo Lu

**Affiliations:** ^1^Henan Academy of Crops Molecular Breeding, Henan Academy of Agricultural Sciences/National Centre for Plant Breeding/Zhengzhou Subcenter of National Soybean Improvement Center/Key Laboratory of Oil Crops in Huanghuaihai Plains of Ministry of Agriculture, Zhengzhou, China; ^2^Soybean Genomics and Improvement Laboratory, USDA-ARS, Beltsville, MD, United States

**Keywords:** soybean, soybean cyst nematode resistance, candidate genes, QTL, whole genome re-sequencing, transcriptome

## Abstract

Soybean cyst nematode (SCN; *Heterodera glycines* Ichinohe) is a highly destructive pathogen for soybean production worldwide. The use of resistant varieties is the most effective way of preventing yield loss. Handou 10 is a commercial soybean variety with desirable agronomic traits and SCN resistance, however genes underlying the SCN resistance in the variety are unknown. An F_2:8_ recombinant inbred line (RIL) population derived from a cross between Zheng 9525 (susceptible) and Handou 10 was developed and its resistance to SCN HG type 2.5.7 (race 1) and 1.2.5.7 (race 2) was identified. We identified seven quantitative trait loci (QTLs) with additive effects. Among these, three QTLs on Chromosomes 7, 8, and 18 were resistant to both races. These QTLs could explain 1.91–7.73% of the phenotypic variation of SCN’s female index. The QTLs on chromosomes 8 and 18 have already been reported and were most likely overlapped with *rhg1* and *Rhg4* loci, respectively. However, the QTL on chromosome 7 was novel. Candidate genes for the three QTLs were predicted through genes functional analysis and transcriptome analysis of infected roots of Handou 10 vs. Zheng 9525. Transcriptome analysis performed also indicated that the plant–pathogen interaction played an important role in the SCN resistance for Handou 10. The information will facilitate SCN–resistant gene cloning, and the novel resistant gene will be a source for improving soybeans’ resistance to SCN.

## Introduction

Soybean cyst nematode (SCN, *Heterodera glycines* Ichinohe) is a devastating pest affecting soybean (*Glycine max* [L.] Merr.) production worldwide (Riggs, 1977; Koenning and Wrather, 2010; Tylka and Marett, 2014; Mitchum, 2016; Miraeiz et al., 2020; Peng et al., 2021). SCN has caused approximately 36% of yield losses in the total soybean production from 1996–2014 in the United States (Kim et al., 2016). In Chifeng area in China, the highest yield reduction rate reached 44.43% because of SCN during 2014–2019 (Li et al., 2020). Moreover, a highly virulent of SCN has been observed in China (Lian et al., 2019). SCN is a soil-borne pathogen and pest management is difficult. SCN management includes crop rotation, pesticide application, biological control, pest-resistant varieties, etc.; however, breeding for resistant varieties is the most effective method (Usovsky et al., 2021). Riggs and Schmitt (1988) reported 16 physiological races that could be differentiated, and the pathogenicity of each race was different. As in Missouri in the United States, the dominant race in the Huanghuai Valley in China is race 2, which was evolved from race 1 (Lu et al., 2006; Mitchum et al., 2007; Lian et al., 2016; Howland et al., 2018). Accordingly, race 2 was used to screen new varieties for SCN resistance in the Huanghuai Valley, unfortunately, most varieties were susceptible to SCN race 2.^1^

Most studies showed that SCN resistance is a quantitative trait and controlled by multiple genes (Concibido et al., 2004; Wang, 2019); more than 200 QTLs have been mapped on 20 chromosomes.^2^ Two major QTLs, *rhg1* (Peking-type *rhg1-a* and PI88788-type *rhg1-b*) and *Rhg4* (*GmSHMT08*), were cloned and functionally analyzed (Cook et al., 2012; Liu et al., 2012; Liu et al., 2017). Three genes (*Glyma.18G022500*, *Glyma.18G022500*, and *Glyma.18G022700*) around the *rhg1-b* locus and the gene *GmSHMT* (*Glyma.08G108900*) around the *Rhg4* locus were identified as major-effect gene to SCN resistance (Cook et al., 2012; Liu et al., 2012; Guo et al., 2020). The PI88788-type requires at least 5.6 copies of *rhg1-b* (Cook et al., 2012; Patil et al., 2019), whereas the Peking-type requires *rhg1-a* and *Rhg4* for SCN resistance (Patil et al., 2019). In the United States, most SCN resistant cultivars are from PI88788 and this has reduced the effectiveness of SCN prevention (Mitchum, 2016). For breeding resistant cultivars, it is vital to identify new quantitative trait loci (QTL) and genes underlying resistance (Liu et al., 2019) and to broaden the genetic basis for improving soybeans’ resistance to SCN. In recent years, Numerous researchers have identified genes (Guo et al., 2020) and reveled a complex regulatory network involved in SCN resistance (Guo et al., 2020; Kofsky et al., 2021; Shi et al., 2021).

Handou 10 was first identified as being resistant against SCN race 1 in routine cultivar testing in 2008 by our team and was registered by the Hebei Variety Approval Committee in 2011. The yield of the variety was significantly better than the control, having the desired agronomic traits in the Hebei uniform test and pre-releasing tests in 2007–2010. However, the origin and inheritance of the SCN resistance in Handou 10 was unknown. The objective of this study was to identify the QTL and candidate genes controlling the resistance and provide the basis for molecular marker development and marker-assisted breeding.

## Materials and Methods

### Plant Materials

A recombinant inbred line (RIL) mapping population of 392 F_2:8_ lines was developed by single seed descent (SSD) from the cross between Zheng 9525 and Handou 10. Zheng 9525 was cultivated by Henan Academy of Agricultural Sciences, whereas Handou 10 was cultivated by Henan Jintun Seed Industry Co., Ltd., and Handan Academy of Agricultural Sciences. The seeds of nine differential cultivars, PI88788, Peking, PI437654, PI209332, PI548316, PI89772, PI90763, Pickett, and Lee, were obtained from Henan Academy of Agricultural Sciences.

### Soybean Cyst Nematode Resistance Identification

Handou 10, Zheng 9525, and nine differential cultivars were evaluated for the resistance to SCN [HG types 2.5.7 (race 1), 1.2.5.7 (race 2), and 1.2.3.5.6.7 (race 4)] in a climate room in Henan Academy of Agricultural Sciences. Plastic cups (Ø 6 cm × h 12 cm) were filled with soil infected by SCN [HG types 2.5.7 (race 1), 1.2.5.7 (race 2), and 1.2.3.5.6.7 (race 4)], respectively. Lees were planted in SCN-infested soil, and after 30 days, we collected cysts from the roots using a 710–250 μm sieve tower. Cysts were collected from the 250 μm sieve and rinsed. The eggs were collected by breaking open cysts with a rubber stopper and collecting the eggs on a sieve stack consisting of 250 μm – 75 μm – 25 μm sieves. The mixture from the 25 μm sieve was backwashed into a 50 mL plastic conical tube. A 40% sucrose solution was added to the tubes, stirred, and centrifuged at 2,000 rpm for 5 min. Eggs in the middle layer or supernatant were then collected over a 25 μm sieve. Handou 10, Zheng 9525, and nine differential cultivars were transplanted with five replicates. Each replicate was one plant in a plastic cup (Ø 6 cm × h 12 cm). Five days after transplantation, seedlings were inoculated with about 4,000 eggs per cup. The plants grew at 70 to 80% relative humidity, 28–24 (L/D), and a photoperiod of 16 h: 8 h (L:D) and were watered daily.

The SCN resistance of the 392 RILs and the parents (Zheng 9525 and Handou 10) were evaluated for SCN resistance against HG type 1.2.5.7 (race 2) and HG type 2.5.7 (race 1) in a climate room. Five days after sowing, two plants of each line were transplanted into a plastic cup (Ø 6 cm × h 12 cm) with three replicates, each replicate had two plants. Five days after transplantation, seedlings were inoculated with about 4,000 eggs per cup. The growth conditions were the same as the above.

Thirty days after inoculation, nematode cysts were collected from the roots of each replicate and counted by an image analysis software (Wang et al., 2014). A female index (FI) was calculated as follows: FI (%) = (average number of cysts on each line/average number of cysts on Lee) × 100. FI was used as phenotype data for QTL analysis. The lines were rated as resistant (FI < 10), moderately resistant (10 ≤ FI < 30), moderately susceptible (30 ≤ FI < 60), or susceptible (FI ≥ 60) to classify the response to SCN.

### DNA Preparation and Whole-Genome Re-sequencing

Leaf samples of each progeny line and their parents were collected at the seedling stage. DNA was extracted by the plant genomic DNA kit [TIANGEN Biotech (Beijing) Co., Ltd.]. Zheng 9525 and Handou 10 were sequenced by Illumina HiSeq 4000, whereas 392 RIL_2:8_ lines from Zheng 9525 × Handou 10 were sequenced by HiSeq X Ten at Huada Gene Technology Co., Ltd. (Shenzhen, China). The sequences of the parents were aligned with the reference genome (Gmax_275_Wm82.a2) using SOAP2 (Li et al., 2009a) and single-nucleotide polymorphisms (SNPs) between the parents were detected by SOAPsnp (Li et al., 2009b). The SNPs between the parents were identified and filtered: (1) Mass value is greater than 20; (2) At least three reads are supported; (3) Heterozygous sites are removed. The pseudomolecules of parental genome sequences were obtained by SNPs between parents with the reference genome. The reads of the progeny population were compared with pseudomolecules of the parental genome sequences using SOAPaligner.

### Genetic Map Construction

Instead of using the SNPs as such for linkage mapping, a sliding window-based approach was used to identify bin markers where consecutive SNPs were merged into one bin. We extract the bin area on each chromosome as bin marker, only the bin markers without segregation distortion are selected to construct a map. A genetic map with the bin markers was constructed with JoinMap 4.0 using the maximum likelihood mapping algorithm (Van Ooijen, 2006). Groups were created depending on LOD scores ≥ 3.0 and a maximum distance of 50 cM. The resulting linkage groups were assigned to specific chromosomes according to the reference genome (Gmax_275_Wm82.a2). Regression mapping was used as the mapping algorithm with Kosambi’s mapping function to convert recombination frequency into map distance.

### Quantitative Trait Loci Mapping

Additive QTLs of SCN resistance with FI to HG types 2.5.7 (race 1) and 1.2.5.7 (race 2), were analyzed using the composite interval mapping (CIM) method in WinQTLCart 2.5 software (Wang et al., 2012). The walking speed for CIM was 1 cM and the LOD threshold at the 5% probability level was determined by a 1,000 permutation test.

### Transcriptome Sequencing

The seedlings with consistent growth were selected to transplant into a plastic cup with sterile soil after Handou 10 and Zheng 9525 were sown in vermiculite, with one plant per cup. Five days later, well-developed plants of the same size were selected to inoculate 4,000 or 0 SCN HG type (1.2.5.7) (race 2) eggs per cup, with three replicates per genotype of each treatment, and five plants for each replicate. Ten days after inoculation, roots were collected, washed, frozen in liquid nitrogen and stored at -80°C until use. Total RNA was extracted by TRIzol (Invitrogen). Each biological replicate contained pooled roots from five individual plants. The RNA transcriptome sequencing and preliminary data analyzes were carried out by Shenzhen Huada Gene Technology Co., Ltd. (China). For each replicate, a mRNA library was constructed and sequenced using the DNBSEQ platform. Adaptor reads, reads with an unknown base N greater than 5%, and low-quality reads (bases with a quality value of less than 15 account for more than 20% of the total bases in the reads) were filtered out of the raw data to obtain high-quality (clean) reads using SOAPnuke (v1.4.0) (Chen et al., 2018). Clean reads were mapped to the soybean reference genome (G.max Wm82.a2.v1) by HISAT (Hierarchical Indexing for Spliced Alignment of Transcripts, V2.1.0) (Kim et al., 2015) and aligned using Bowtie 2 (v2.2.5) (Langmead and Salzberg, 2012). The genes and transcripts were calculated using RSEM (v1.2.8) (Li and Dewey, 2011). Significant differentially expressed genes (DEGs) were obtained with false discovery rates (FDRs) of ≤ 0.05.

### Functional Annotation and Pathway Enrichment

Significant DEGs were annotated with Kyoto Encyclopedia of Genes and Genomes (KEGG).^3^ The statistical enrichment of DEGs in KEGG pathways was accomplished with the R (version 3.1.1) function phyper.^4^ Then FDR was performed on *p*-value, and *q*-values of ≤ 0.05 was considered to be significantly enriched.

### Candidate Genes Analyzes of Major Quantitative Trait Loci Intervals

The QTLs that could be detected for the resistance of Handou 10 to SCN HG type 2.5.7 (race 1) and 1.2.5.7 (race 2) were considered major QTLs. The physical positions of the major QTL intervals were identified according to the above genetic map and the reference genome (“Williams 82.a2.v1”) (Jiang et al., 2018). The DEGs between the infected roots of Handou 10 and Zheng 9525 in the major QTL intervals were the candidate genes controlling SCN resistance in Handou 10.

### KASP Markers Analysis

DNA was extracted by the plant genomic DNA kit [TIANGEN Biotech (Beijing) CO., Ltd]. DNA concentration and quality were measured with NanoDrop 2000 (Thermo Fisher Scientific, Waltham, MA, United States). According to the SNPs between Zheng 9525 and Handou 10, the KASP (Kompetitive allele-specific PCR) markers were designed by LGC Science (Shanghai) Ltd. The primer sequences of KASP markers linked to *rhg1* and *Rhg4* were referred to the reports from Shi et al. (2015) and Kadam et al. (2016). The PCR reaction mixture was prepared according to the instructions of the KBioscience (Herts, United Kingdom). The program was set to hold at 94°C for 15 min, followed by 10 touch-down cycles of 20 s at 94°C and 1 min at 65–59°C (dropping 0.6°C per cycle), and then 23 cycles of 20 s at 94°C, 1 min at 57°C. The PCR amplification product was read with a PHERAstar SNP typing detector, and SNP alleles were determined based on the ratio of fluorescence signals.

## Results

### Identification of Soybean Cyst Nematode Resistance

**FIGURE 1:**
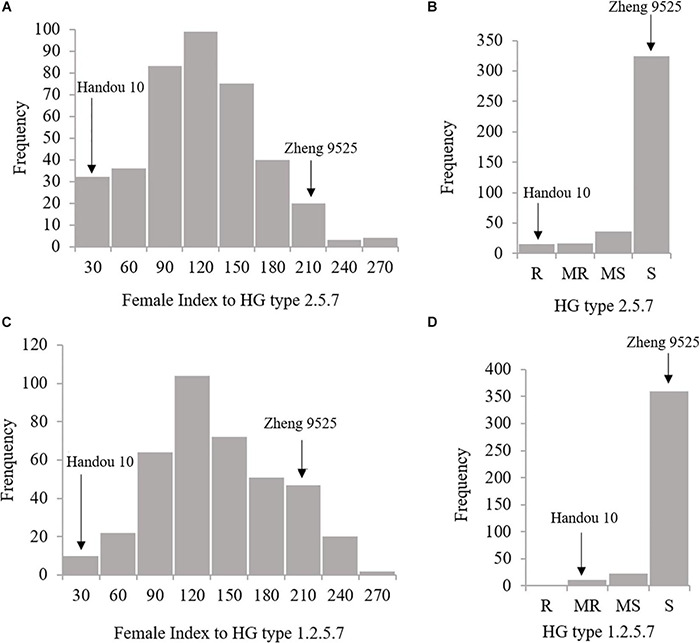
Female index (FI) and rating distributions of SCN resistance to HG type 2.5.7 and HG type 1.2.5.7 for the RILs derived from Zheng 9525×Handou 10, respectively. **(A)** The FI distribution of SCN resistance to HG types 2.5.7; **(B)** the rating of SCN resistance to HG types 2.5.7; **(C)** the FI distribution of SCN resistance to HG types 1.2.5.7; **(D)** the rating of SCN resistance to HG types 1.2.5.7.

Handou 10, Zheng 9525, and nine differential cultivars (with Lee as a susceptible control) were screened for SCN resistance. Handou 10 was resistant to SCN HG type 2.5.7 (race 1), moderately resistant to SCN HG type 1.2.5.7 (race 2) and moderately susceptible to HG type 1.2.3.5.6.7 (race 4) (Table 1). Among the 392 RILs, 3.83, 4.34, 9.18, and 82.65% were resistant, moderately resistant, moderately susceptible and susceptible to race 1, respectively, whereas 2.55, 5.61, and 91.84% of individuals were moderately resistant, moderately susceptible and susceptible to race 2, respectively. The FI ranged from 0.16 to 253.80%, and 13.87 to 245.05% for HG type 2.5.7 (race 1) and HG type 1.2.5.7 (race 2), respectively (Figure 1 and Table 2). None of the 392 RILs was resistant to SCN HG type 1.2.5.7 (race 2) (Figure 1).

**TABLE 1:** Evaluation of Handou 10, Zheng 9525, and eight differential cultivars for resistance to three HG types of soybean cyst nematode (SCN).

Varieties	SCN HG type					
	2.5.7 (race 1)		1.2.5.7 (race 2)		1.2.3.5.6.7 (race 4)	
	FI (%)	Rating*	FI (%)	Rating*	FI (%)	Rating*
Peking§	5.6	R	37.2	MS	70.1	S
PI 88788	37.7	MS	99.5	S	87.2	S
PI 90763	0.6	R	5	R	65.9	S
PI 437654	1.1	R	1.4	R	2.7	R
PI 209332	42.4	MS	98.1	S	55.8	MS
PI 89772	1.3	R	6.8	R	47.9	MS
PI 548316	53.2	MS	91.5	S	99.5	S
Handou 10	6.3	R	28	MR	53.3	MS
Zheng 9525	107.2	S	114.6	S	83.7	S
Pickett	2.5	R	58.5	MS	77.9	S

*: R: Resistant, 0 < FI ≤ 10%; MR: medium resistance, 10 < FI ≤ 30%; MS: medium susceptibility, 30 < FI ≤ 60%; S: susceptible, FI > 60. §: PI 548402.

**TABLE 2:** Descriptive statistics of the female index (FI) of the parents and the 392 F_2:8_ RILs from Zheng 9525 × Handou10 after inoculation of SCN HG types 2.5.7 (race 1) and 1.2.5.7 (race 2), respectively.

HG type	Parent		RILs					
	Handou 10	Zheng 9525	Mean	Min	Max	Std. Error	Skewness	Kurtosis
2.5.7 (race 1)	0.86	198.57	125.34	0.16	253.80	50.25	0.23	−0.49
1.2.5.7 (race 2)	18.61	192.35	103.82	13.87	245.05	49.54	0.15	0.06
								

### Analysis of Whole-Genome Re-sequencing

Zheng 9525 and Handou 10 were re-sequenced using an Illumina HiSeq4000 platform with sequencing average depths of 11.94× and 16.94×, respectively (Supplementary Table 1). Approximately 79.25 and 111.92 M reads, and 11.84 and 16.74 G bases of raw data were obtained from the two parents, respectively; a total of 78.25 reads from Zheng 9525 and 110.09 M reads from Handou 10 were mapped, and the mapped bases of each genotype were 11.74 and 16.64 G, respectively. The genome coverage of these two cultivars was 91.77 and 93.43%, respectively (Supplementary Table 1). A total of 1,062,100 SNPs between Zheng 9525 and Williams 82, and a total of 946,194 SNPs between Handou 10 and Williams 82 were identified using SOAPsnp, and a total of 732,008 SNPs could be identified between the parents.

The average sequencing depth of 392 RILs was 1.94× and the coverage rate was 62.38% (Supplementary Table 2). The sequencing data showed that the average mapped reads was 12.74 M and the average bases were 1.91 G. A total of 607,635 SNPs were identified in the RIL population and 8,593 breaking points were detected using the sliding window approach (Supplementary Table 3).

### Construction of High-Density Genetic Map

A high-density genetic map was constructed by joining 5,233 bin markers (missing < 20%, Supplementary Table 4). The map comprised 4763.23 cM with an average distance of 0.91 cm between adjacent markers (Table 3). Chromosome 18 had the highest number of bin markers (405 markers). The linkage map length was the largest for chromosome 5 (325.35 cM), and the smallest was for chromosome 4 (89.47 cM) (Table 3).

**TABLE 3:** Distribution of polymorphic bins for each chromosome of the 392 RILs mapping population of the cross Zheng 9525 × Handou 10.

Chromosome	No. of bins	Map length (cM)	Ave interval (cM)	Chromosome	No. of bins	Map length (cM)	Ave interval (cM)
1	311	149.51	0.48	11	103	189.13	1.84
2	197	198.87	1.01	12	214	229.84	1.07
3	236	223.21	0.95	13	337	281.09	0.83
4	151	89.47	0.59	14	305	295.35	0.97
5	277	325.35	1.17	15	151	250.53	1.66
6	237	242.65	1.02	16	307	295.30	0.96
7	284	324.35	1.14	17	385	199.82	0.52
8	318	308.13	0.97	18	405	199.61	0.49
9	285	267.91	0.94	19	198	277.57	1.40
10	159	122.34	0.77	20	373	293.21	0.79

### Quantitative Trait Loci Mapping

Seven QTLs for SCN resistance with FI were detected on six chromosomes by CIM, which were designated as *SCN_7_1*, *SCN_8_2*, *SCN_12_3*, *SCN_15_4*, *SCN_18_5*, *SCN_18_6*, and *SCN_20_7*, respectively (Table 4). The resistance alleles of five QTLs (*SCN_7_1*, *SCN_8_2*, *SCN_18_5*, *SCN_18_6*, and *SCN_20_7*) were derived from the resistant parent Handou 10, and the resistance alleles of the remaining two QTLs (*SCN_12_3* and *SCN_15_4*) were derived from the susceptible parent. Three QTLs (*SCN_7_1*, *SCN_8_2*, and *SCN_18_6*) were detected in race 1 and race 2. The percentage of the explained variance of the identified QTLs varied from 1.91 to 7.73%. According to their physical position in the genome, the QTL intervals of *SCN_8_2* (6.75–10.35 Mb) and *SCN_18_6* (0.05–3.16 Mb) overlapped with the *rhg1* and *Rhg4* loci (see Footnote 2), respectively.

**TABLE 4:** Summary of additive QTLs for SCN resistance detected in the mapping population of 392 RILs derived from the cross between susceptible parent Zheng 9525 and resistant parent Handou 10 using composite interval mapping (CIM) with the female index of HG types 2.5.7 (race 1) and 1.2.5.7 (race 2).

QTL	Chromosome	Physical position (Mb)	Map position (cM)	Peak (cM)	LOD	R^2^ (%)	Additive effect	HG type
SCN_7_1	7	16.55–18.45	180–189	187	1.99	1.90	7.08	1.2.5.7
		17.85–19.15	184–201	192	4.91	4.72	11.27	2.5.7
SCN_8_2	8	6.75–10.05	86–106	99	5.07	4.93	11.11	1.2.5.7
		7.15–10.35	89–108	103	5.23	5.06	11.38	2.5.7
SCN_12-3	12	7.55–7.65	79–83	83	2.19	2.05	-7.17	1.2.5.7
SCN_15-4	15	14.95–15.35	69–71	70	2.25	2.13	-7.40	2.5.7
SCN_18-5	18	51.95–53.85	2–17	9	2.08	2.27	7.50	1.2.5.7
SCN_18-6	18	0.05–3.15	178–199	187	5.61	7.73	14.41	1.2.5.7
		0.05–3.15	178–200	185	3.13	3.98	10.42	2.5.7
SCN_20-7	20	45.15–45.75	288–291	290	2.27	2.17	7.32	1.2.5.7

*FI: female index. Physical position was based on the Glycine max genome assembly version Wm82.a2v1. The significant LOD thresholds were estimated by 1,000 permutations at the 5% significance level as follows: 1.79 for HG type 1.2.5.7 and 2.49 for HG type 2.5.7. R^2^: percentage of phenotypic variance explained by a QTL. Additive effect: The positive value implies that Handou 10 decreases the phenotypic value (FI); the negative value implies that Zheng 9525 decreases the phenotypic value.*

### Transcriptomic Analysis

**FIGURE 2:**
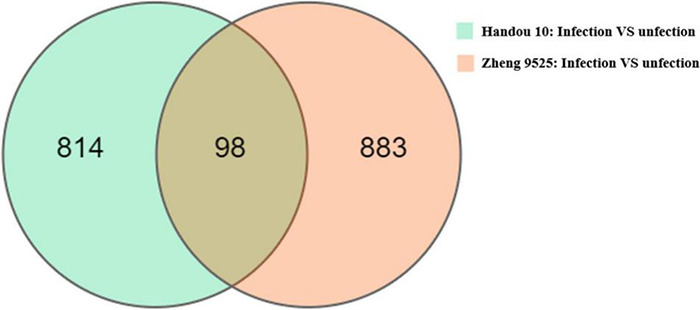
Different expressed genes (DEGs) in Handou 10 and Zheng 9525. S: Zheng 9525 with SCN infected; R: Handou 10 with SCN infected; CK_R: mock-inoculated control from Handou 10; CK_S: mock-inoculated control from Zheng 9525.

We analyzed the transcriptomes of the resistant and susceptible parents infected with race 2. Each sample produced 6.45 G data on average (Supplementary Table 5). The average comparison rate between each sample and reference genome was 80.70%. A total of 912 and 981 significant DEGs were detected between the infected and uninfected roots in Handou 10 and Zheng 9525, respectively (Figure 2). In Handou 10, 312 and 600 up- and down- regulated genes were detected, respectively. In Zheng 9525, 395 and 586 up- and down- regulated genes were detected, respectively. A total of 4424 DEGs were detected between the infected roots of Handou 10 and Zheng 9525.

**FIGURE 3:**
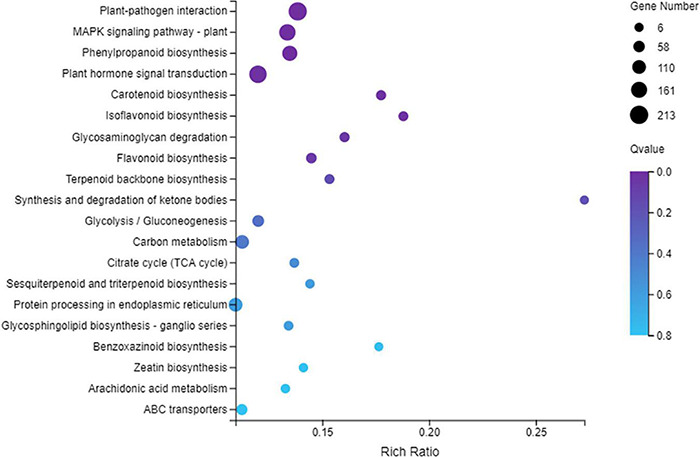
Statistics of KEGG Pathway Enchrichment scatter diagram of DEGs between infected roots of Handou 10 and Zheng 9525. The 20 most enriched pathways are displayed.

The KEGG analysis showed that 2100 DEGs were assigned to 132 pathways, and the plant–pathogen interaction pathway (ko04626) was the top enriched pathway (Figure 3) with 213 DEGs between the SCN infected roots of Handou 10 and Zheng 9525. The results suggested that the plant–pathogen interaction played the most important role in the SCN resistance for Handou 10. MPK signaling-plant (ko04016), phenylpropanoid biosynthesis (ko00940), plant hormone signal transduction (ko04075) and other pathways also played important roles in the resistance to SCN (Figure 3).

### Candidate Gene Analyzes of SCN_7_1, SCN_8_2, and SCN_18_6

According to the physical positions of the major QTL intervals, there were 6, 23, and 12 candidate genes from the DEGs between the infected roots of Handou 10 and Zheng 9525 for *SCN_7_1*, *SCN_8_2*, and *SCN_18_6*, respectively (Table 5). The candidate gene *Glyma.08G108900* at *SCN_8_2* and candidate genes *Glyma.18G022400, Glyma.18G022500*, and *Glyma.18G022700* at *SCN_18_6* have been reported for SCN resistance (Guo et al., 2019; Cook et al., 2012; Liu et al., 2012). *Glyma.08G108900* was located in the *Rhg4* locus. *Glyma.18G022400*, *Glyma.18G022500*, and *Glyma.18G022700* were located in the *rhg1* locus. These three genes (*Glyma18g02580*, *Glyma18g02590*, and *Glyma18g02610* in Wm82.a1) in *rhg1* were all expressed in the resistance of Handou 10 according to the transcriptome data (Table 5). The genotypes of KASP markers linked to *rhg1* (Rhg1-2, Rhg1-5, DD381, and DD383) and *Rhg4* (Rhg4-3, Rhg4-5, and DD191) for Handou 10 were the same as those in Peking, whereas the genotypes of those (DD7_1, DD7_5, DD7_8, DD7_9, DD7_11, and DD7_16) located at *SCN_7_1* for Handou 10 were consistent with those in PI437654 (Table 6 and Supplementary Table 6). There is no relevant report about the candidate genes at *SCN_7_1* and these are likely novel.

**TABLE 5:** Candidate genes at three QTLs (*SCN_7_1*, *SCN_8_2*, and *SCN_18_6*).

Gene ID	log_2_ (R/S)	FDR(S-vs-R)	Annotations
*Glyma.07G139700*	3.34	8.31E-12	Glutathione S-transferase
*Glyma.07G139800*	1.30	2.74E-04	Glutathione S-transferase
*Glyma.07G143500*	-3.01	8.31E-07	Leucine-rich repeat protein
*Glyma.07G151500*	1.14	5.36E-03	Glutaryl-CoA dehydrogenase
*Glyma.07G152900*	1.82	2.49E-09	DNAJ homolog subfamily C member
*Glyma.07G154200*	1.62	6.64E-03	Kelch repeat domain
*Glyma.08G097200*	2.66	9.44E-04	FMN-dependent dehydrogenase
*Glyma.08G097300*	-5.79	1.64E-25	peroxisomal (S)-2-hydroxy-acid oxidase GLO3
*Glyma.08G100500*	1.06	7.44E-03	F-box domain
*Glyma.08G101000*	-2.87	5.27E-08	Transferase family
*Glyma.08G104200*	1.07	9.68E-03	Rubber elongation factor protein (REF)
*Glyma.08G106200*	-1.64	2.02E-03	Amidase family protein
*Glyma.08G108800*	1.48	4.55E-09	Adenosylhomocysteinase
*Glyma.08G108900*	1.91	4.87E-09	Serine hydroxymethyltransferase
*Glyma.08G109100*	2.45	1.15E-04	NAD dependent epimerase/dehydratase
*Glyma.08G110300*	4.41	3.09E-09	Chalcone and stilbene synthase family protein
*Glyma.08G114400*	-2.12	4.97E-04	Chlorophyllide a oxygenase
*Glyma.08G116900*	2.18	2.95E-03	Cysteine protease family C1-related
*Glyma.08G118900*	2.35	4.99E-19	Glutathione S-transferase
*Glyma.08G119200*	-1.44	7.66E-04	Disease resistance protein
*Glyma.08G120500*	-2.67	1.50E-03	Oligopeptide transporter-related
*Glyma.08G122200*	-1.85	2.46E-17	ATP-dependent protease
*Glyma.08G123200*	1.00	7.05E-03	Pterin-4-alpha-carbinolamine dehydratase
*Glyma.08G125600*	-2.42	1.96E-09	UDP-glucuronosyl and UDP-glucosyl transferase
*Glyma.08G125900*	2.79	8.34E-26	Hypothetical protein glysoja_015556
*Glyma.08G128900*	-1.40	7.35E-05	Leucine rich repeat receptor-like protein kinase
*Glyma.08G129600*	1.22	1.18E-09	Cationic amino acid transporter
*Glyma.08G131900*	-2.62	4.62E-03	Integral component of membrane
*Glyma.08G134900*	-1.12	3.71E-04	F-box and associated interaction domains-containing protein
*Glyma.18G008600*	-1.33	9.39E-05	Protein of unknown function (DUF3537)
*Glyma.18G012700*	-2.76	4.61E-19	Multidrug/pheromone exporter, ABC superfamily
*Glyma.18G018200*	1.44	2.46E-05	AP2 domain
*Glyma.18G018500*	2.64	3.50E-04	Pollen proteins Ole e I like
*Glyma.18G018600*	1.04	6.43E-03	Myo-inositol-1-phosphate synthase
*Glyma.18G020400*	1.14	2.67E-03	Zinc finger domain-containing
*Glyma.18G020500*	2.32	3.05E-03	Ring finger domain-containing
*Glyma.18G022400*	1.66	5.19E-09	Amino acid transporter
*Glyma.18G022500*	1.62	3.67E-27	soluble NSF attachment protein (SNAP)
*Glyma.18G022600*	2.02	2.90E-04	Protein of unknown function (DUF2985)
*Glyma.18G022700*	1.51	7.58E-10	Wound-induced protein WI12
*Glyma.18G028200*	-1.82	6.55E-11	Nucleoporin NUP84-related
			

**TABLE 6:** Detection and analysis of soybean germplasm by KASP markers located at major *QTL* intervals (*SCN_8_2*, *SCN_18_6*, and *SCN_7_1*).

DNA \Assay	Rhg1-2^a^	Rhg1-5^a^	GSM381^b^	GSM383^b^	Rhg4-3^a^	Rhg4-5^a^	GSM191^b^	DD7_1^C^	DD7_5^C^	DD7_8^C^	DD7_9^C^	DD7_11^C^	DD7_16^C^
Handou 10	GG	CC	GG	GG	TT	GG	GG	AA	TT	TT	AA	TT	AA
Zheng 9525	CC	GG	TT	CC	AA	CC	CC	CC	AA	AA	GG	CC	CC
PI437654	GG	CC	GG	GG	AA	GG	GG	CC	TT	TT	AA	TT	AA
Peking	GG	CC	GG	GG	TT	GG	GG	AA	AA	AA	GG	CC	AA

*^a^Genotypes of four KASP markers adapted from Kadam et al. (2016). ^b^Genotypes of three KASP markers adapted from Shi et al. (2015). ^c^Genotypes of six KASP markers developed from SCN_7_1.*

## Discussion

Handou 10 is a variety with a broad–spectrum resistant to SCN. Our recent experiment result showed that Handou 10 was resistant to SCN HG type 7 (race 3). In this study, Handou 10 was identified as being resistant against HG type 2.5.7 (race 1), and moderately resistant against HG type 1.2.5.7 (race 2); additionally, its resistance was identified as being Peking-type based on the analysis of the QTL (Table 4), KASP markers (Table 6) and the CNV at the *rhg1*/*Rhg4* (Supplementary Figure 1). *SCN_8_2* and *SCN_18_6* overlapped with the resistance loci *Rhg4* and *rhg1-a*, respectively. This two additive QTLs to SCN resistance with FI were also detected by IciMapping 4.2 software (Supplementary Table 7). SCN_8_2 and SCN_18_6 were interactive for SCN resistance to HG types 2.5.7 (race 2) (Supplementary Table 8). According to the CNV analyzes (Suvakov et al., 2021) for the whole-genome re-sequencing, the CNV at the *rhg1* and *Rhg4* for Handou 10 is 2.95 and 2.05, respectively (Supplementary Figure 1). The resistance to SCN and CNV at the *rhg1*/*Rhg4* in Handou 10 is similar to PI404166, PI 437679, and PI089772 (Patil et al., 2019). The *rhg1-a*, sometimes in combination with the *Rhg4*, provides strong resistance to SCN (Guo et al., 2019). Three genes (*Glyma.18G022400, Glyma.18G022500*, and *Glyma.18G022700*) are responsible for the resistance provided by *rhg1-b* (Cook et al., 2012*).* In our study, these three candidate genes in *rhg1-a* might be regulate the SCN resistance of Handou 10. However, the mechanisms of *rhg1-a* and *rhg1-b* to SCN resistance are not same (Cook et al., 2012; Patil et al., 2019). For *rhg1-b*, overexpression of the individual genes in roots was ineffective and SCN resistance is conferred by copy number variation. The Peking-type requires *rhg1-a* and *Rhg4* for SCN resistance (Patil et al., 2019). In this study, the expression of *Glyma.08G108900* located in the *Rhg4* locus was verified by qPCR (Supplementary Figure 2) and this was basically consistent with transcriptome analysis. *Glyma.08G108900* was responsible for the resistance to SCN and the function has been verified. The resistant mechanism of Handou 10 is complicate and we are breeding new superior lines with it now.

*SCN_7_1* is an important QTL to SCN resistance for Handou 10. Some QTLs (Webb et al., 1995; Ferreira et al., 2011; Abdelmajid et al., 2014; Chang et al., 2016; Vuong et al., 2015; Li et al., 2016) related to SCN resistance on chromosome 7 were reported at www.soybase.org. The physical location of *SCN_7_1* was basically the same as the marker php02301a mapped in PI437654 (Webb et al., 1995), but far from the QTLs detected by other researchers (see Footnote 2). There are six DEGs in the *SCN_7_1* region and these candidate genes are not previously described as SCN resistance genes. Among the DEGs at *SCN_7_1*, *Glyma.07G139700* and *Glyma.07G139800* both code Glutathione S–transferases (GSTs), which were up–regulated between infected and uninfected SCN in Handou 10 and down–regulated in Zheng 9525 according to transcriptome data. Recently, the expression of *Glyma.07G139800* was verified by qPCR (Supplementary Figure 2) and this was basically consistent with transcriptome analysis in our study. GSTs are multifunctional enzymes which play a crucial role in cellular detoxification and oxidative stress tolerance (Rezaei et al., 2013). GST was elevated in the SCN–infected roots relative to uninoculated roots (Alkharouf et al., 2004). The over-expression of a GST gene from wild soybean (Glycine soja) enhances drought and salt tolerance in transgenic tobacco (Ji et al., 2010). However, the relationship between glutathione metabolism and the disease resistance of Handou 10 still needs to be further studied.

Soybean resistance to SCN is regulated by multiple genes. In our study the plant-pathogen interaction pathway was the most enriched KEGG pathway between infected Handou 10 and Zheng 9525, and between infected Zheng 9525 and uninfected Zheng 9525, whereas the MAPK signaling pathway was the most enriched KEGG pathway between infected and uninfected Handou 10. Plants and pathogens should be studied together as an interacting system (Peyraud et al., 2017). Soybean root cells undergo dramatic morphological and biochemical changes after being infected with SCN (Khan et al., 2004). The development of cyst nematodes in the infected SCN roots in Handou 10 was slower than in Zheng 9525 after 10 days of inoculation (Supplementary Figure 3). Many DEGs may be related to the development of soybean cyst nematode. These DEGs genes (*Glyma.07G139700*, *Glyma.07G139800, Glyma.08G108900*, *Glyma.08G118900*, *Glyma.08G119200, Glyma.18G022400*, *Glyma.18G022500*, and *Glyma.18G022700)* might be the important candidate gene to SCN resistance according to KEGG analysis and genes function.

## Data Availability Statement

The datasets presented in this study can be found in online repositories. The names of the repository/repositories and accession number(s) can be found below: NGDC CAR005982.

## Author Contributions

WL, JW, and HW conceived the project and designed the experiments. HW, YL, JL, HL, YW, CL, SW, and HZ performed the experiments. HW, WL, JW, and QS participated in the data analysis and manuscript revised. All authors contributed to the article and approved the submitted version.

## Conflict of Interest

The authors declare that the research was conducted in the absence of any commercial or financial relationships that could be construed as a potential conflict of interest.

## Publisher’s Note

All claims expressed in this article are solely those of the authors and do not necessarily represent those of their affiliated organizations, or those of the publisher, the editors and the reviewers. Any product that may be evaluated in this article, or claim that may be made by its manufacturer, is not guaranteed or endorsed by the publisher.

## Footnotes

1
http://www.zzj.moa.gov.cn

2
https://www.soybase.org

3
https://www.genome.jp/kegg/

4
https://en.wikipedia.org/wiki/Hypergeometric_distribution
