# Supplementary material for: Identification of Candidate Genes Controlling Soybean Cyst Nematode Resistance in “Handou 10” Based on Genome and Transcriptome Analyzes
Source: Front Plant Sci. 2022 Mar 15;13:860034. doi: 10.3389/fpls.2022.860034 (PMC8965568; doi:10.3389/fpls.2022.860034)
Supplement: Supplementary file 1 [file Data_Sheet_1.doc]

Supplementary Material

**Supplementary Figures**


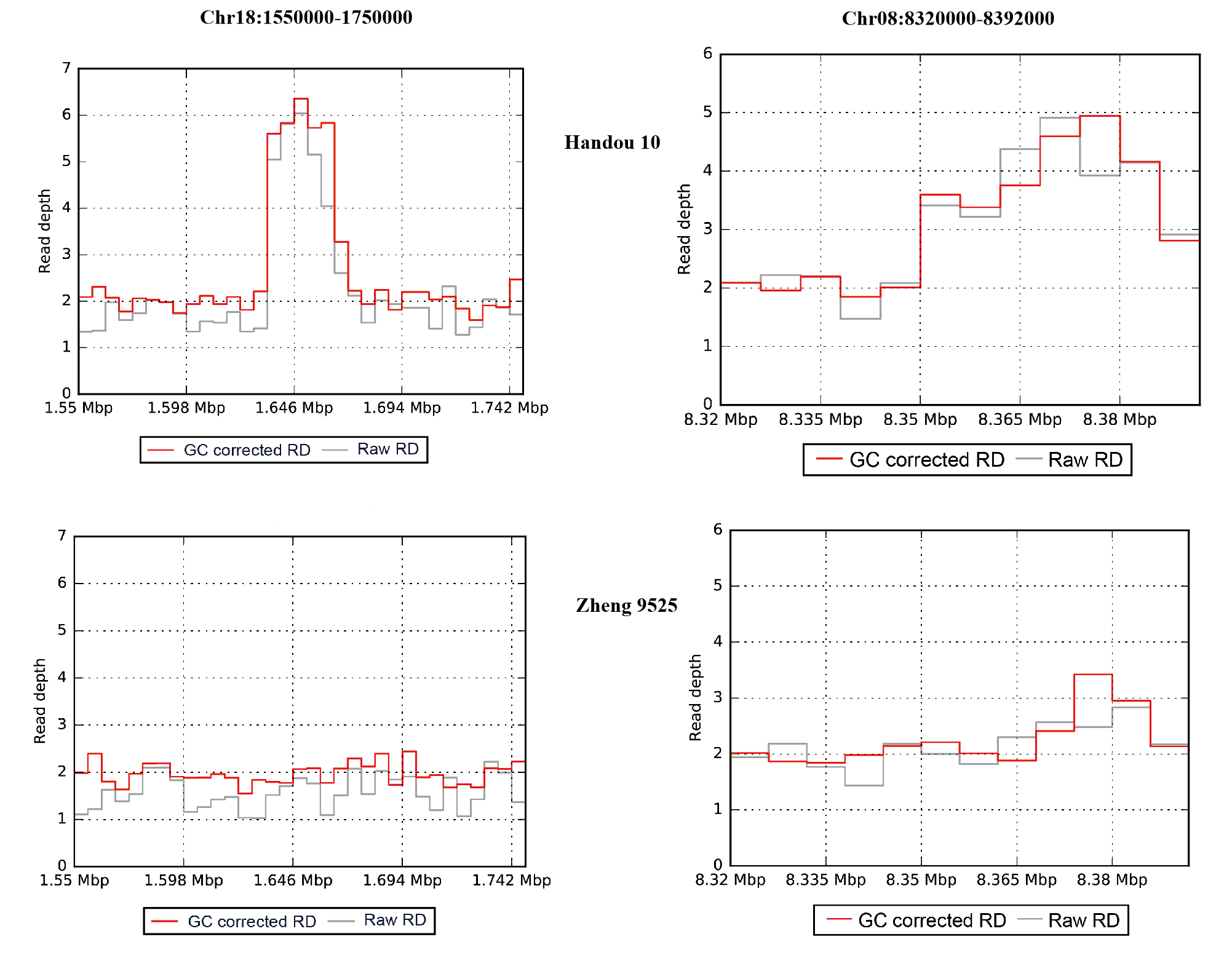


Figure S1. Copy number variation (CNV) of the *rhg1* and *Rhg4* locus defined from whole-genome re-sequencing for Handou 10 and Zheng 9525. CNV analysis was performed using coverage-based CNVpytor (a python extension of CNVnator) to determine copy number of *rhg1* and *Rhg4* (Suvakov et al., 2021).


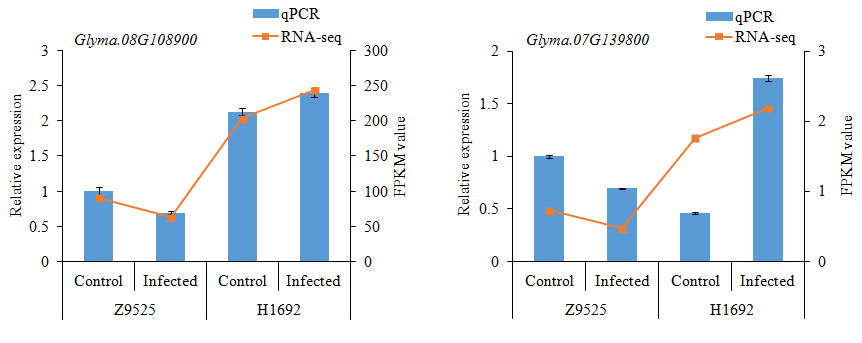


Figure S2. Expression levels of the two candidate genes. Blue is the expression level of *Glyma.08G108900* and *Glyma.07G139800* by quantitative real-time PCR. Orange is the FPKM value of *Glyma.08G108900* and *Glyma.07G139800* by RNA-seq.


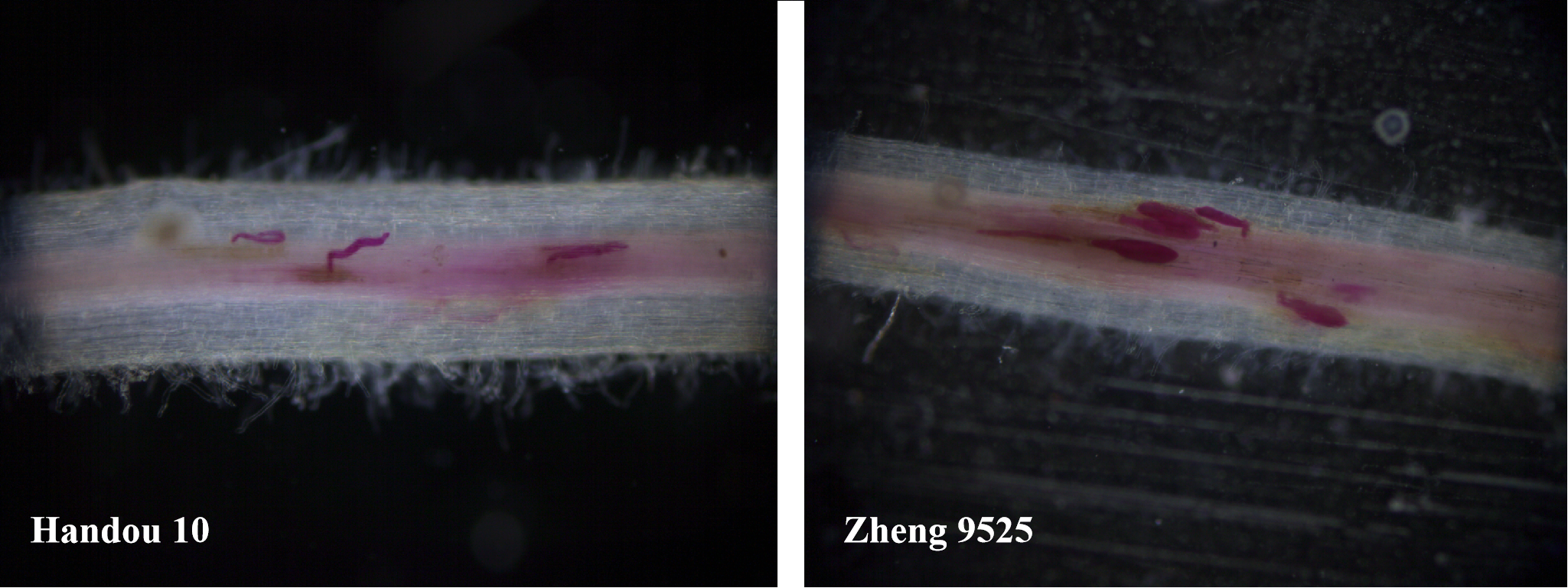


Figure S3. Representative SCN-infested roots from Handou 10 and Zheng 9525 after 10 days inoculation, respectively. Root vascular cylinder and nematode were stained with acid fuchsin.
